# A Reinforcement Learning Approach to View Planning for Automated Inspection Tasks

**DOI:** 10.3390/s21062030

**Published:** 2021-03-13

**Authors:** Christian Landgraf, Bernd Meese, Michael Pabst, Georg Martius, Marco F. Huber

**Affiliations:** 1 Fraunhofer Institute for Manufacturing, Engineering and Automation IPA, Nobelstraße 12, 70569 Stuttgart, Germany; michael.pabst@ipa.fraunhofer.de (M.P.); marco.huber@ieee.org (M.F.H.); 2 Max Planck Institute for Intelligent Systems, Max-Planck-Ring 4, 72076 Tübingen, Germany; georg.martius@tuebingen.mpg.de; 3 Institute of Industrial Manufacturing and Management IFF, University of Stuttgart, Allmandring 35, 70569 Stuttgart, Germany

**Keywords:** view planning, reinforcement learning, simulation, robotics, smart sensors, automated inspection

## Abstract

Manual inspection of workpieces in highly flexible production facilities with small lot sizes is costly and less reliable compared to automated inspection systems. Reinforcement Learning (RL) offers promising, intelligent solutions for robotic inspection and manufacturing tasks. This paper presents an RL-based approach to determine a high-quality set of sensor view poses for arbitrary workpieces based on their 3D computer-aided design (CAD). The framework extends available open-source libraries and provides an interface to the Robot Operating System (ROS) for deploying any supported robot and sensor. The integration into commonly used OpenAI Gym and Baselines leads to an expandable and comparable benchmark for RL algorithms. We give a comprehensive overview of related work in the field of view planning and RL. A comparison of different RL algorithms provides a proof of concept for the framework’s functionality in experimental scenarios. The obtained results exhibit a coverage ratio of up to 0.8 illustrating its potential impact and expandability. The project will be made publicly available along with this article.

## 1. Introduction

### 1.1. Motivation

Due to the lack of skilled workforce, quality and productivity aspects, as well as cost advantage, the importance of robotics and automation in production has grown significantly in recent years [1]. Industrial robot manipulators are extremely versatile and dominate most manufacturing processes and inspection procedures [2]. Fast and easy programming of new tasks is a key challenge to enable efficient and profitable use of robot technology, especially in case of small lot sizes. Despite its intuitive and concise operation, *online* programming via teach pendants is a time-consuming and tedious procedure, and only economically viable in case of large lot sizes. Hence, *offline* alternatives allowing for a straightforward implementation of new inspection tasks are gaining attention in industrial research.

Offline programming (OLP) systems are based on CAD models and robot simulation software. OLPs generate robot programs without interrupting production and fully exploit knowledge from CAD assemblies and planning algorithms. However, a not precisely reproduced real-world setting without a strong reference to the online setup with its robot leads to discrepancies between simulation and real-world and requires costly post-processing. Manipulators equipped with sensors such as 3D stereo cameras can automatically inspect assemblies and avoid manual post-processing of robot programs.

Therefore, the goal of this works consists of developing an intelligent framework to automatically generate suitable robot view poses for inspection based on a CAD model. It allows the integration of any workpiece providing the availability of its CAD model, any robot and sensor supported in ROS and any RL algorithm available in the commonly used libraries. The work is meant to pave the way for automated inspection and CAD-based robot programming.

### 1.2. Related Work

View pose generation for inspection tasks can be subdivided into two groups: In the case of reconstructing unknown or free form objects, the next best view pose is estimated after each measurement [3,4]. This procedure, commonly called the *next best view* problem, has not been approached in the context of the present work. On the other hand, CAD model-based *view pose planning* enables prior planning of all required view poses and is the focus of this paper. The (offline) search for a set of view poses is known as the view planning problem (VPP) and was described comprehensively by [5]. Beyond that, Ref. [6] provides a general survey on VPP and active vision development.

The VPP is a geometric problem and can be modeled mathematically as an NP-hard set cover problem (SCP), which has been surveyed for decades [7]. Assuming the availability of a CAD model of the particular workpiece, an early approach by [8] established a measurability matrix indicating the visibility of a finite set of surface points linked to a finite set of viewpoints. This concept was extended in [3] by adding further constraints to the measurability matrix and proposing a two-stage approach including a rough and fine modeling phase. Although the work by [8] suggested simulated annealing, Ref. [3] implemented a greedy search algorithm, Ref. [9] proposed an evolutionary search algorithm and [10] applied linear programming to solve the VPP. As outlined by [11], these methods lack performance gains and efficiency over simple greedy algorithms.

Reinforcement learning (RL) is a more recent approach for solving SCP-related optimization problems and has exhibited remarkable results in other areas [12]. The work of [11] identifies an RL workflow using three different RL algorithms including on-policy SARSA, Temporal Difference (TD), as well as off-policy Q-learning in the context of VPP. We deployed a comparable off-policy Q-learning as used by [11] in the presented framework to demonstrate its general functionality. Furthermore, Ref. [13] implemented an ε-greedy-based approach for online viewpoint generation in a robotic inspection scenario, which can be seen in the spirit of previously mentioned *next best view* scenario. In the past 10 years, more sophisticated, value-based RL algorithms have been developed: Ref. [14] presented Deep Q-Networks (DQN), where deep learning function approximation is introduced for the action-value function estimation. Since DQN was even further improved regarding its systematic overestimation of real Q-values (Double DQN [15]) and sample inefficiency (DQN with Prioritized Experience Replay [16]) it is also integrated in the presented OLP framework for solving the VPP.

Although these value-based, model-free RL algorithms are suited for determining view poses in discrete action spaces, their major drawback is a consequence of a fundamental assumption of the VPP itself. The assumption, that a close-to-perfect set of view poses can be achieved by a proper subset of a finite number of predefined actions (to view poses), is usually not covered by real-world state/action spaces. Although increasing the discrete number of predefined poses is a reasonable approach to extenuate the problem, it also entails an increasing computational effort.

To avoid the illustrated VPP drawback, one has to bypass the problem of discrete action spaces. Although methods using policy gradients such as REINFORCE [17] tend to converge fast and can be applied to problems with any type of actions, including continuous action spaces, they lack in sample efficiency. Therefore, a hybrid form of value- and policy-based methods are widely used when dealing with continuous action spaces, so-called actor-critic methods. The release of Asynchronous Advantage Actor-Critic (A3C) [18] had a big impact on RL with multiple asynchronous, in parallel trained agents exploring large state-action spaces in comparatively less time. The next breakthrough, Proximal Policy Optimization (PPO) by [19], significantly stabilized the training of actor-critic methods by using a clipping function that limits the policy update per training step. PPO has gained lots of attention, is still among state-of-the-art actor-critic approaches at the time of writing and therefore the third applied method to find suitable sets of view poses.

Recently, Ref. [20] presented *robo-gym*, a similar open-source approach to train RL algorithms on both simulated and real-world robots. It might be interesting to compare both frameworks in detail although *robo-gym* is not yet fully available for ROS Noetic and Python 3 and not specifically designed for sensor simulation and view planning.

To execute any VPP solution on both real or simulated robots, collision-free robotic paths need to be planned that do not suffer from singularities and are optimized in terms of time and accuracy. This path planning problem is closely related to the Traveling Salesman Problem (TSP), which optimizes the cost of consecutive tasks, e.g., by restructuring its order. In literature, the combination of VPP and TSP is considered to be Coverage Planning Problem (CPP) or more specificall Robotic Task Sequencing Problem (RTSP). However, we use the common planning algorithms from the Open Motion Planning Library (OMPL) [21] for path planning and focus on VPP.

### 1.3. Contribution

In this work, we present a holistic approach for finding high-quality view pose sets for 3D surface inspection of a given workpiece using a robot and 3D sensor in combination with the workpiece’s CAD model. The novel framework automates view planning in inspection tasks for any industrial robot arm available in ROS and any 3D sensor specification (resolution, working distance, etc.) with a close link to the real-world setup.

The second major achievement consists of transferring the latest RL-based concepts to the domain of VPPs and proposing a generic mathematical formulation. The approach enables the direct application of state-of-the-art RL methods (DQN, PPO) and straightforward integration of additional methods available in the OpenAI libraries. We evaluate the view planning system for different workpieces from the ABC dataset [22] as well as a custom assembly to demonstrate its effective operation. Our procedure reduces the programming time for robotic inspection tasks and increases the quality and efficiency at the same time.

A key point to emphasize is that the complete code along with installation instructions and video footage is available at https://github.com/christianlandgraf/rl_viewplanning (accessed on 12 March 2021) and may serve as starting point for other RL-based view planning experiments.

### 1.4. Structure

The article is structured as follows. In Section 2, we describe the used robot cell and sensor setup. All individual components for dataset integration, sensor simulation, path planning, and Reinforcement Learning of the framework are introduced and explained. The experimental results of the proposed framework are presented in Section 3. We investigated three RL algorithms as well as different workpieces for inspection. Section 4 elaborately discusses the findings and proposes potential improvements. At last, Section 5 wraps up our contributions and provides a prospect of future work.

## 2. Proposed Architecture (Methods)

The following section introduces the chosen setup comprising a 3D sensor attached to a robot arm and its corresponding simulation modules. The subsequent sections describe each component of the framework, namely robot environment, task environment, and learning algorithm. Briefly summarized, the learning algorithm level implements a specific RL algorithm. On top of that, the task environment explicitly formulates actions, states and reward specific to view planning. The robot environment builds a bridge to the simulation modules.

### 2.1. Hardware Setup

**Figure 1:**
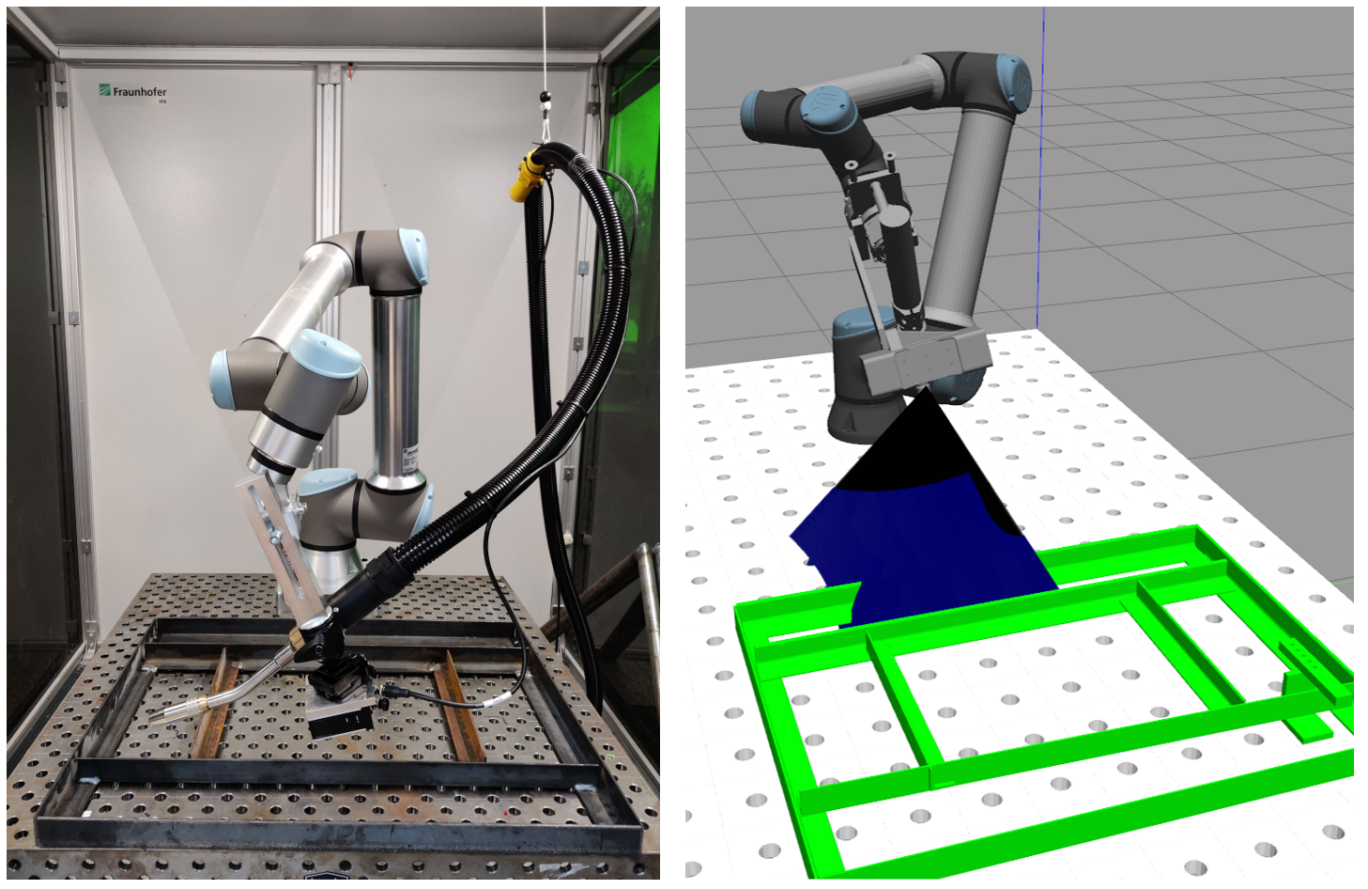
Exemplary robot cell in real-world (**left**) and simulation (**right**).

The experimental setup consists of a collaborative Universal Robots UR10e robot equipped with an Ensenso N35 3D sensor as an endeffector. The UR10e possesses six degrees of freedom, a reach of 1300 mm, and a pose repeatability of ±0.05 mm. Ensenso N35-606-16-BL is a 3D stereo projection sensor using blue light. It has a clearing distance (CD) of 312 mm and a working distance (WD) of up to 464 mm. The sensor has a resolution of 1280 × 1024 Pixel (1.3 MP) and a focal length of 6 mm. This corresponds to a spatial (*x-y*) resolution of 0.383 mm/pixel at a WD of 400 mm. The *z*-axis accuracy of the Ensenso N35 at 400 mm WD is 0.192 mm. Figure 1 illustrates the hardware setup in real-world and its simulated equivalent.

### 2.2. Simulation

#### 2.2.1. Controller Simulation

**Figure 2:**
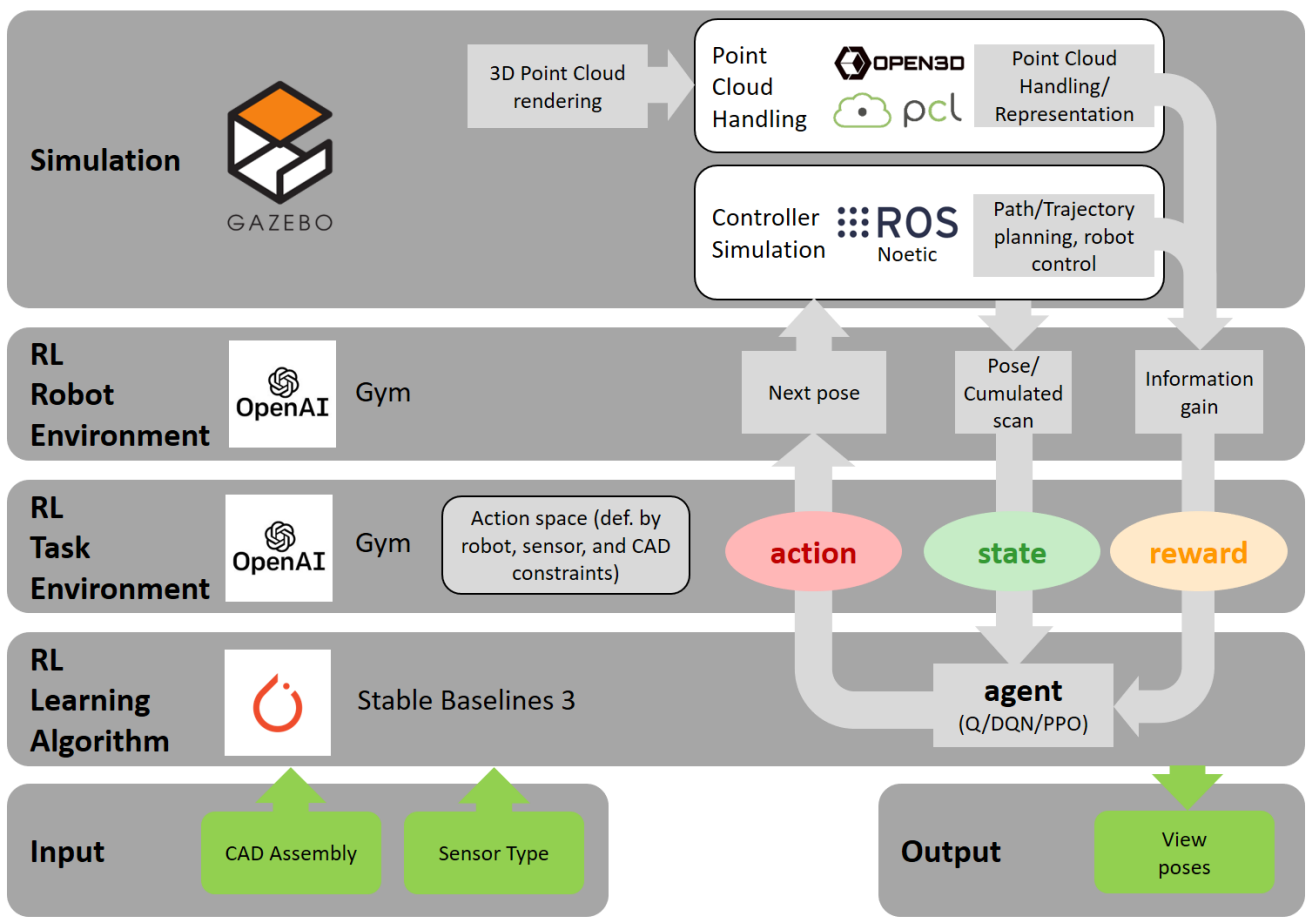
The framework architecture separated by application layer. Each instance of a layer inherits its upper layer and displays a one-to-many relationship, e.g., multiple RL task environments descend from a robot environment.

Figure 2 shows the overall architecture of the simulation and Reinforcement Learning environment. The framework builds on top of the OpenAi ROS toolkit [23]. Starting at the lowest layer, we choose Gazebo [24] as simulation software due to the existing feature of rendering realistic 3D stereo camera data and its close link to the ROS Noetic middleware. Other robot simulations as *MuJoCo*, *Blender*, *CoppeliaSim*, or *Webots* either lack in point cloud rendering or in less developed ROS support. The common controller plugins of *ros_control* [25] executes planned robot paths to view poses on the robot and can seamlessly switch between real-world and simulation.

#### 2.2.2. Pointcloud Handling

**Figure 3:**
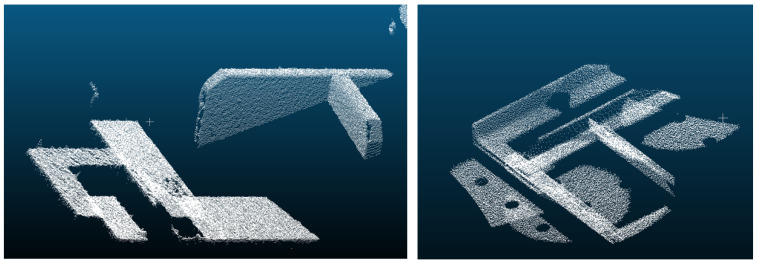
A real point cloud taken by an Ensenso N35 (**left**) and a simulated pointcloud (**right**).

The point cloud rendering is based on the velodyne_simulator ROS package (https://bitbucket.org/DataspeedInc/velodyne_simulator, visited on 25 January 2021) and supports Gaussian noise and GPU acceleration. Figure 3 illustrates its realism. Since Gazebo simulates a hardware interface to the ROS robot driver (in our case, Universal Robots), superior layers work independently of choosing a real robot or its simulated counterpart. The same applies to the Gazebo sensor simulation and the Ensenso ROS sensor driver. Further point cloud processing and semantic evaluation is based on Point Cloud Library (PCL) [26] and Open3D [27] as described in Section 2.3.2.

### 2.3. Reinforcement Learning

#### 2.3.1. Robot Environment

The robot environment layer provides an interface between a specific task environment and a common robot cell simulation. Proposed actions of the RL agent are translated into according robot movements using MoveIt [28], which offers a ROS platform for OMPL and collision detection libraries. To accelerate learning procedures, we optionally neglect detailed path planning and immediately set the sensor origin to the desired poses. Kinematic and reachability constraints must be checked individually or covered during presampling of potential view poses. A detailed overview of performance in terms of training speed is given in Section 3.

#### 2.3.2. Task Environment

Depending on the specific scenario, the task environment takes the current robot pose and corresponding information gain by a point cloud measurement, assembles observations, shapes the reward, translates actions and implements stop criteria. In our case, we parametrize a task environment for VPPs allowing different families of RL agents, action and observation spaces and predefined constraints on view poses. This is presented in detail in the subsequent paragraphs.

Next, an RL agent operates above the task environment and learns to predict high-quality view poses. Since the simulation environment implements the required methods of OpenAI gym [29], theoretically, any RL algorithm in the OpenAI baselines library can be used. Due to its ongoing support and PyTorch interface, we only tested the Stable Baselines 3 fork [30]. It is possible to start and parallelize multiple environments simultaneously to speed up training.

Besides the detailed parametrization on task environment and learning algorithm level, the user needs to choose a workpiece and its approximate pose as input and define proper sensor characteristics. We integrate an exemplary subset of the ABC dataset [22] and a custom test workpiece for experiments in Section 3.

In the following paragraphs, we formulate the briefly described components of the task environment in detail, based on the mathematical foundation.

#### Theoretical Background

A Reinforcement Learning problem consists of an *agent*, which performs *actions* inside an *environment* and learns from its observed *states* and derived *rewards* or *penalties*, respectively [12]. Mathematically, this is expressed by a Markov Decision Process (MDP). MDPs are assembled by four components: a state $s_{t}\in S$, where *t* determines the current time step; an action $a_{t}\in A$; a transition probability $P(s_{t+1}|s_{t},a_{t})$ from state $s_{t}$ to another state $s_{t+1}$ depending on the selected action $a_{t}$; and a carefully constructed reward $R_{t}\left(s_{t+1}|s_{t},a_{t}\right)$. Due to its nature of merging state transitions, MDPs satisfy the Markov Property such that all previous states $s_{1},\ldots ,s_{t-1}$ are represented by the most recent state $s_{t-1}$.

A policy $\pi_{\theta }\left(a_{t}|s_{t}\right)$ represents the decision making process of choosing an action $a_{t}$ at state $s_{t}$ and with parameters θ. The common goal of RL methods consists of finding an optimal decision process. In practice, the environment model is unknown. Therefore, most approaches either use a *value-based* or *policy-based* approach, or a combination of both to learn from interaction with the environment. As indicated by its name, *value-based* approaches aim at optimizing a value function $v_{\pi }\left(s\right)$, which predicts the maximum expected reward $R_{t}$ for a given state $s_{t}$ [12]. The value function is defined as

(1) $$v_{\pi }\left(s\right)=E_{\pi }\left[\sum_{k=0}^{\infty }\gamma^{k}R_{t+k+1}|s_{t}=s\right],$$

where γ∈[0,1) denotes a discount factor to balance short-term and long-term rewards. In contrast, *policy-based* RL methods directly optimize the policy function $\pi_{\theta }$. These methods are better suited for continuous action spaces, but suffer from sample inefficiency.

MDPs are a subset of the more generalized definition of Partially Observable Markov Decision Processes (POMDP) [12]. Agents in POMDPs do not possess knowledge of the complete environment state space but rather construct states based on observations. The subsequent approach for RL-based view planning will build the state space similarly.

#### Action and State

In our view planning approach, an action consists of choosing a view pose and subsequently planning and executing the robot movement toward this pose. As soon as the robot reaches its goal, the sensor renders a 3D point cloud at this pose. The environment state is constructed from the observations consisting of 3D measurements and current robot pose.

Q-learning and DQN are based on a finite number of actions, which corresponds to a set of potential view poses arranged across the workpiece. We implemented a tool for the individual adjustment of a view pose grid including its geometry (triangular/squared), density in *x*-, *y*-, and *z*-directions as well as the sensor orientation as roll, pitch and yaw angles. In the following, we define all coordinates with respect to the default world coordinate system. We further set the sensor origin such that the *x*-axis is pointing out of its lens and use the roll (*R*), pitch (*P*), and yaw (*Y*) angle definition. For setting up the framework, step sizes $s_{x}$, $s_{y}$, and $s_{z}$ are to be chosen. The sensor orientation requires step sizes $n_{R}$, $n_{P}$, and $n_{Y}$ that result in corresponding numbers of step $n_{R}$, $n_{P}$ and $n_{Y}$.

We use the bounding box center $c=(x_{c},y_{c},z_{c})$ of the workpiece as well as its width $x_{wp}$, length $y_{wp}$, and a threshold ε, to define the action space expansion in the *x-y*-plane. The height limits of the sensor are chosen according to its working range, i.e., above its clearing distance $\left(z_{\mathrm{dist}\_\min}\right)$ and within the scanning range $\left(z_{\mathrm{dist}\_\max}\right)$. Next, we specify a starting position $\left(x_{0},y_{0},z_{0}\right)$ at one corner and a corresponding limit for the *x*, *y*, and *z* values at the opposite corner $\left(x_{\lim},y_{\lim},z_{\lim}\right)$:

(2) $$\left(\begin{array}{c} x_{0}\\ y_{0}\\ z_{0} \end{array}\right)\;=\;\left(\begin{array}{c} x_{c}-\frac{x_{wp}}{2}-\varepsilon \\ y_{c}-\frac{y_{wp}}{2}-\varepsilon \\ z_{c}+z_{\mathrm{dist}\_\min} \end{array}\right),\;\left(\begin{array}{c} x_{\lim}\\ y_{\lim}\\ z_{\lim} \end{array}\right)\;=\;\left(\begin{array}{c} x_{c}+\frac{x_{wp}}{2}+\varepsilon \\ y_{c}+\frac{y_{wp}}{2}+\varepsilon \\ z_{c}+z_{\mathrm{dist}\_\max} \end{array}\right).$$

Based on the starting position $\left(x_{0},y_{0},z_{0}\right)$, the action space is defined by iteratively adding the step sizes until we exceed the opposite limit $\left(x_{\lim},y_{\lim},z_{\lim}\right)$. Equation (3) formally defines the action space $A_{1}$ consisting of the view pose grid.

(3) $$A_{1}=\left\{\left(x,y,z,R,P,Y\right)\in \left(\begin{array}{c} \left\{x=x_{0}+i\cdot s_{x}\;|\;i\in N,x<x_{\lim}\right\}\\ \left\{y=y_{0}+i\cdot s_{y}\;|\;i\in N,y<y_{\lim}\right\}\\ \left\{z=z_{0}+i\cdot s_{z}\;|\;i\in N,z<z_{\lim}\right\}\\ \left\{R_{init}+\left(i-1\right)\cdot s_{R}\;|\;i\in 1,\ldots ,n_{R}\right\}\\ \left\{P_{init}+\left(i-1\right)\cdot s_{P}\;|\;i\in 1,\ldots ,n_{P}\right\}\\ \left\{Y_{init}+\left(i-1\right)\cdot s_{Y}\;|\;i\in 1,\ldots ,n_{Y}\right\} \end{array}\right)\right\}.$$

**Figure 4:**
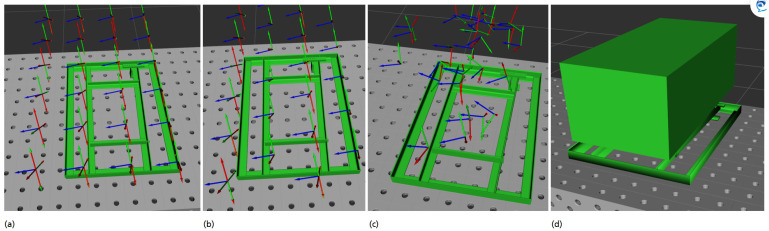
Sampling discrete actions (poses, respectively) in (**a**) a squared grid or (**b**) in a triangular grid with four sensor orientations per position or (**c**) randomly inside a continuous space. Figure (**d**) depicts a continuous action space.

Similarly, we define a second view pose grid $A_{2}$, where the *y* direction is shifted by $\frac{s_{y}}{2}$ in every second step, which is intended to prevent inaccessible blind spots between view poses. An example of a triangular view pose grid is shown in Figure 4.

In addition to lattice-like structures for view poses, we also evaluate a random sampling of view poses as done by [11,13]. Therefore, we use the previously defined limits $\left(x_{0},y_{0},z_{0}\right)$ and $\left(x_{\lim},y_{\lim},z_{\lim}\right)$ to construct a box. To increase sample efficiency and avoid empty point clouds, the sensor orientation points towards the workpiece. View poses out of the robot’s reach are rejected during sampling, too. The RL algorithm learns to choose a qualified set of view poses among the samples. Equation (4) defines this action space named $A_{3}$.

(4) $$A_{3}=\left\{\left(x,y,z,R,P,Y\right)\in \left(\begin{array}{c} \left[x_{0},\;x_{\lim}\right]\\ \left[y_{0},\;y_{\lim}\right]\\ \left[z_{0},\;z_{\lim}\right]\\ \left[0,\;360\right]\\ \left[210,\;330\right]\\ \left[0,\;360\right] \end{array}\right)\right\}.$$

For policy-based algorithms such as PPO, we define a continuous instead of a discrete action space with a finite number of poses. The action space extends across a similar cuboid used for the grid with the same boundaries for *x*, *y*, and *z* used for discrete action spaces. Instead of proposing a number between 1 and the number of view poses in the finite set, the action is now represented by a pose within predefined limits:

(5) $$A_{4}\;=\;\left(\begin{array}{c} \left[x_{0},\;x_{\lim}\right]\\ \left[y_{0},\;y_{\lim}\right]\\ \left[z_{0},\;z_{\lim}\right]\\ \left[0,\;360\right]\\ \left[210,\;330\right]\\ \left[0,\;360\right] \end{array}\right).$$

Figure 4 shows examples for discrete action spaces with fixed *z* value as well as a continuous action space. The observation space $o_{t}$ is constructed by the current sensor position, the information gain from this step ($I_{t}$) and the cumulated point cloud of all sensor measurements of this episode ($pc_{cum}$) (see Equation (6)).

(6) $$o_{t}=\left(x_{sensor},y_{sensor},z_{sensor},R_{sensor},P_{sensor},Y_{sensor},I_{t},pc_{cum}\right)$$

The information gain is subject of the next paragraph. The actual state of the environment is simply represented by the current sensor pose as defined in Equation (7). The current point cloud is omitted since it would increase the state’s memory size dramatically. Therefore, the state is constructed as follows:

(7) $$s=(x_{sensor},y_{sensor},z_{sensor},R_{sensor},P_{sensor},Y_{sensor}).$$

We will discuss the consequences and alternatives of dropping point cloud measurements in Section 4. To avoid negative implications during our experiments, we prevent the RL agent from approaching the same or very similar poses multiple times on task-level.

#### Reward

The reward of each step is based on the scanned, previously unseen surface area. Mathematically, we express this as the set-theoretic difference of the surface area scan $SA_{t}$ at state *t* and the episode’s cumulated scan $A_{cum,t-1}$, which both are normalized to the workpiece total surface area $SA_{total}$. However, convenient and established triangulation methods for point cloud surface reconstruction could not be used, since they either tend to wrap around noisy surface scans more or less doubling its surface area or are too computing-intensive. Therefore, we developed a custom return-module that is optimized in terms of accuracy and speed using the PCL library [26].

To obtain the covered surface area, we smooth each initial scan (t=1) for noise reduction. Although this step is not crucial for training with simulated point clouds, it is required for processing of real-world point clouds. A second step converts the point cloud to a voxel grid of size 0.0015 m. The covered surface area $SA_{t}$ is approximated by the multiplication of voxel count and $0.0015^{2}$ and normalized to the workpiece’s total surface area, $SA_{total}$. Finally, we export the processed voxel grid as the first part of the cumulating point cloud.

Since the voxel size limits the minimal thickness of potential objects to 0.0015 m, one may reduce its size if required, providing a sufficiently precise real-world robot and sensor setup.

For any subsequent scan (t>1), we first subdivide the previously scanned, cumulated point cloud $pcd_{t-1,cum}$ into $pcd_{t-1,in}$ and $pcd_{t-1,out}$ based on the bounding box of the current scan $pcd_{t}$ for faster point cloud processing. Then the normalized surface areas of $pcd_{t-1,in}\left(SA_{t-1,in}\right)$ and of the point cloud merge of $pcd_{t-1,in}$ and $pcd_{t}\left(SA_{t,merge}\right)$ are calculated as described above. The normalized surface area gain for state $s_{t}$ is the difference of both and is similar to the total area gain of $s_{t}$. Finally, the merged point cloud $pcd_{t,merge}$ inside the bounding box is merged with $pcd_{t-1,out}$ and exported as new cumulated point cloud $pcd_{t,cum}$. Equation (8) formulates the proposed reward $R_{t}$ at time step *t*.

(8) $$R_{t}=\left\{\begin{array}{cc} SA_{t}/SA_{total}, & \mathrm{if}\;t=\;1\\ \left(SA_{t}\backslash SA_{t-1,cum}\right)/SA_{total}, & \mathrm{if}\;t>\;1 \end{array}\right.\;\in \left[0,1\right]$$

#### 2.3.3. Learning Algorithm

To evaluate the presented method, we approached the VPP using three different algorithms. First, we applied Q-learning along the lines of [11,23]. Second, we deployed DQN [14] on a similar discrete pose set and PPO [19] using a continuous state/action space.

In off-policy Q-learning [31], the objective is to optimize Q-function $Q^{*}$ by learning Q-values for each state-action pair $\left(s_{t},a_{t}\right)$ within the discrete action spaces. Therefore, it is necessary to find the maximum expected future reward for each possible state-action pair to select the best action by a given state. The Q-learning equation consists of the old action-value function $Q(s_{t},a_{t})$, the reward $R_{t+1}$ after taking action $a_{t}$, a learning rate α>0, and the discounted expected future reward $\gamma \max_{a}Q\left(s_{t+1},a\right)$:

(9) $$Q^{*}\left(s_{t},a_{t}\right)\leftarrow Q\left(s_{t},a_{t}\right)+\alpha \left(R_{t+1}+\gamma \max_{a}Q\left(s_{t+1},a\right)-Q\left(s_{t},a_{t}\right)\right).$$

During training, the Exploration Rate ϵ controls whether an action is chosen based on prior experience or randomly. It balances the exploration of unknown states and the exploitation of gained knowledge and decays each episode through the Exploration Discount Factor. Additionally, a lower limit for the exploration rate $\epsilon_{\min}$, avoiding an imbalance between exploration and exploitation [12].

However, off-policy Q-learning assumes that all states and actions are stored (e.g., in a Q-table), which becomes infeasible when it is applied to real-world problems. We are limited in finding good view poses and there might be much better view poses, which cannot be learned because of the limited state and action space. Nevertheless, solving the VPP with Q-learning as done by [11] is not the goal of this work. Off-policy Q-learning will serve as a comparison benchmark to highlight the benefits of other RL approaches.

To avoid this issue, we also applied DQN with experience replay as proposed by [14]. The core of the used DQN architecture is a multi-layer perceptron with 2 layers with 64 neurons. The deep neural network is trained with a mini-batch gradient descent optimization [14]. DQN approximates the Q-function using mini-batches for training and returns actions with the highest expected reward for any input state. The objective consists of minimizing a cost function based on the network weights θ to approach the Q-function. Equation (10) describes the learning process. The neural network weights θ are iteratively updated through

(10) $$\theta^{*}\leftarrow \theta_{t}+\alpha \left[R_{t+1}+\gamma \max_{a}Q\left(s_{t+1},a;\theta \right)-Q\left(s_{t},a_{t};\theta \right)\right]\nabla {}_{\theta }Q\left(s_{t},a_{t};\theta \right){|}_{\theta =\theta }\;,$$

where $\theta^{*}$ are the desired network weights the Q-Net is converging to. The reward term is similar to Q-learning, except that *Q* also depends on the network weights θ. Finally, $\nabla {}_{\theta }Q\left(s_{t},a_{t};\theta \right)$ is the gradient of the loss function obtained through back propagation and used to update the network weights.

**Figure 5:**
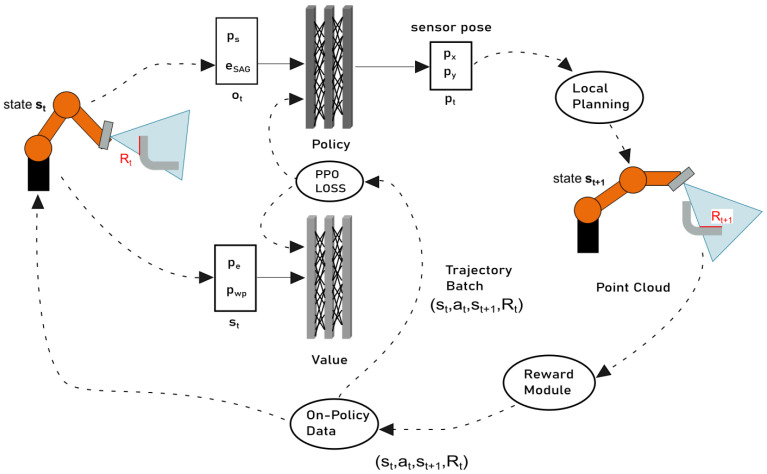
PPO approach for view planning. Here, $e_{SAG}$ represents the surface area gain, $p_{s}$ the sensor pose, $p_{wp}$ the current workpiece pose, $p_{e}$ the current robot pose and $p_{t}={\left(p_{x},p_{y}\right)}^{T}$ the selected sensor pose.

Finally, we integrated PPO [19] as an RL approach that is applicable on continuous action spaces. Figure 5 illustrates the structure of the PPO approach to view planning and is now explained in detail.

A main advantage of PPO compared to other Actor-Critic methods is that the policy update is clipped guaranteeing monotonic policy improvement and therefore a very robust training. This is accomplished by PPO’s *clipped surrogate objective*

(11) $$L^{CLIP}\left(\theta \right)={\hat{E}}_{t}\left[\min\left(r_{t}\left(\theta \right){\hat{A}}_{t},clip\left(r_{t}\left(\theta \right),1-\varepsilon ,1+\varepsilon \right){\hat{A}}_{t}\right)\right].$$

Here, the objective function includes the conservative policy gradient objective $r_{t}\left(\theta \right){\hat{A}}_{t}$. This estimator consists of the probability ratio $r_{t}\left(\theta \right)$ and the estimator ${\hat{A}}_{t}$ of an advantage function and the clipped version of the conservative policy gradient objective $clip\left(r_{t}\left(\theta \right),1-\varepsilon ,1+\varepsilon \right){\hat{A}}_{t}$ using the hyperparamter ε, defining the clipping range. Equation (11) is optimized over a batch of samples, which is indicated by the expectation ${\hat{E}}_{t}$.

The agent is trained using the loss function in (12), which contains the objective from (11) and two additional terms:

(12) $$L_{t}^{PPO}\left(\theta \right)={\hat{E}}_{t}\left[L^{CLIP}\left(\theta \right)-c_{1}L_{t}^{VF}\left(\theta \right)+c_{2}S\left[\pi_{\theta }\right]\left(s_{t}\right)\right].$$

where $L_{t}^{VF}\left(\theta \right)$ is a squared-error loss, *S* denotes an entropy bonus, and $c_{1}$ and $c_{2}$ are the loss value function and loss entropy coefficients, respectively. Typical for Actor-Critic methods, parameters are shared between the policy and value neural networks. Therefore, a correlation between the policy objective $L^{CLIP}\left(\theta \right)$ and a value error term $c_{1}L_{t}^{VF}\left(\theta \right)$ is considered in (12) besides the exploration error term $c_{2}S\left[\pi_{\theta }\right]\left(s_{t}\right)$, which checks if the exploration frequency is high enough. The link of the PPO architecture with policy and value networks, operating as actor and critic, to the task environment of the presented framework is visualized in Figure 5.

## 3. Experiments and Results

**Figure 6:**
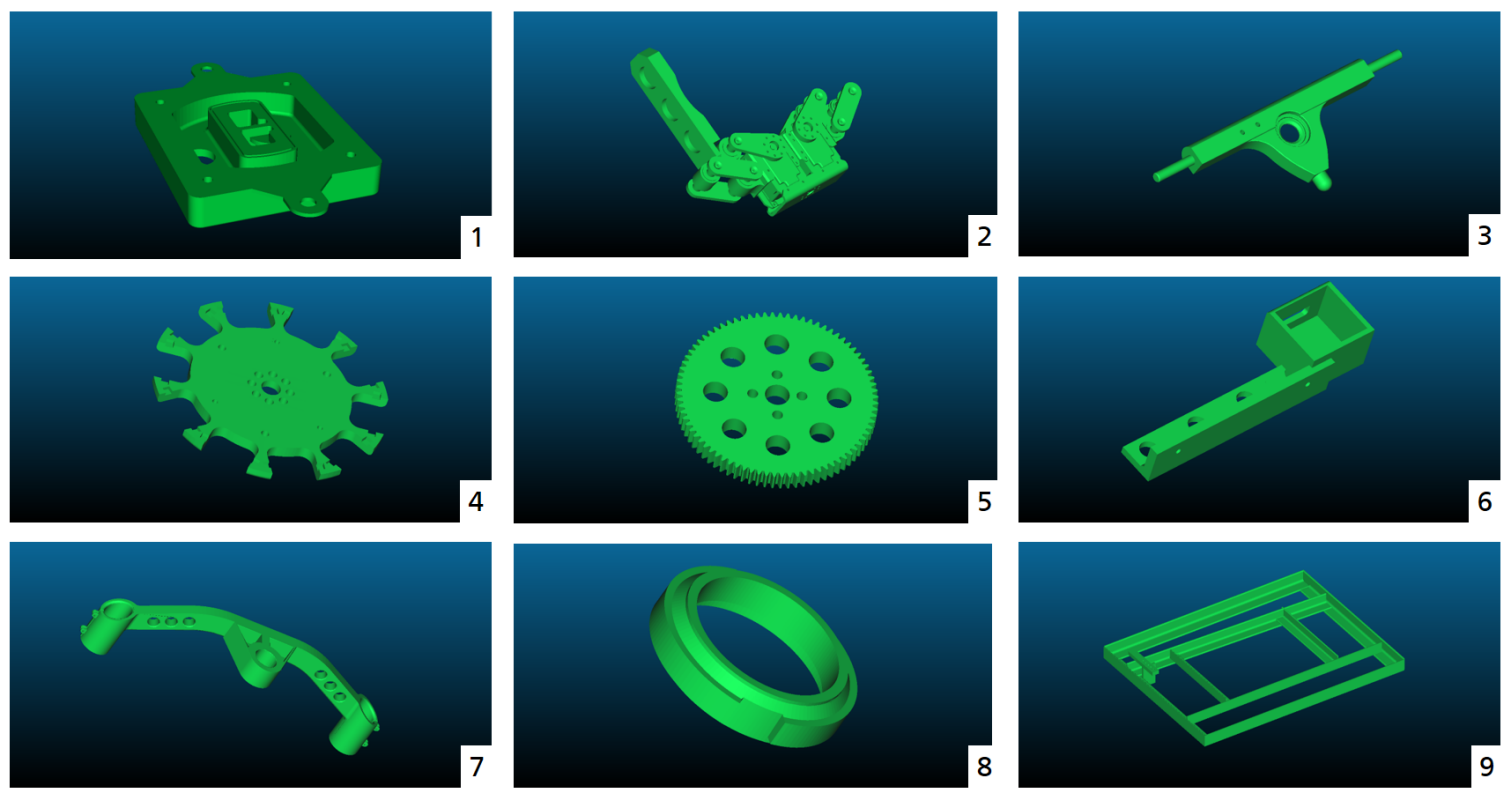
An illustration of the test workpieces used in our experiments. Each of them is part of the ABC Dataset [22], except for the custom workpiece number 9.

This section presents various experiments with the newly introduced learning framework for view pose planning to provide a proof of concept. Figure 6 shows the exemplary integrated test workpieces from the open-source ABC dataset [22], which collects about one million models in total, as well as a custom test workpiece. The workpieces were scaled such that they are approximately the same size.

We present experimental results for the three RL algorithms introduced in Section 2.3.3. Table 1a displays the training settings for Q-learning, Table 1b for DQN and Table 1c for PPO. The experiments have been executed on a PC with 32 GB RAM, an Intel Xeon W-2125 processing unit with 8 cores and 4 GHz clock rate, and a Nvidia Quadro P2000 GPU with 32 GB. The simulation framework achieves about 3 steps per second. Each iteration takes 0.38 s. However, the actual performance heavily depends on the desired sensor resolution (see Section 2.1) and whether one wants to simulate the actual execution of the robot path. More specifically, the reward calculation takes about 0.11 s, the sensor placement about 0.052 s, and the sensor measurement and conversion to the correct format about 0.25 s.

To deploy grid-like structures in case of a discrete action space (see Section 2.3.3, Equation (3)), the step sizes $s_{x}$ and $s_{y}$ in *x* and *y* direction are set to 0.2 m. Due to the small working range of the simulated Ensenso N35 sensor, the sensor height $z_{0}$ is equal to 0.3 m, i.e., $z_{\mathrm{dist}\_\min}\;=\;z_{\mathrm{dist}\_\max}\;=\;0.3$. Since rotation around the x-axis results in negligible changes of the resulting point cloud, the roll angle *R* remains fixed $\left(R_{0}=0\right)$. The pitch angle is set to a fixed value of $255^{{}^{\circ}}$. The step size of the yaw angle is set to $90^{{}^{\circ}}$ starting from $45^{{}^{\circ}}$, such that the action space considers four different yaw angles $45^{{}^{\circ}}/135^{{}^{\circ}}/225^{{}^{\circ}}/315^{{}^{\circ}}$. The resulting grid contains 36 positions with four different orientations at each position, making up a total of 144 view poses for both triangle and square grids.

To construct an action space of randomly chosen view poses, we sample about 70 poses as described in Equation (4), again with a fixed *z*-offset of 0.3 m.

The continuous actions space for PPO is constructed using similar limits for *x*, *y*, and *z* following Equation (5). We use fixed initial values for *z*, *R*, *P*, and *Y* to reduce the dimensionality of the action space and facilitate learning

**Figure 7:**
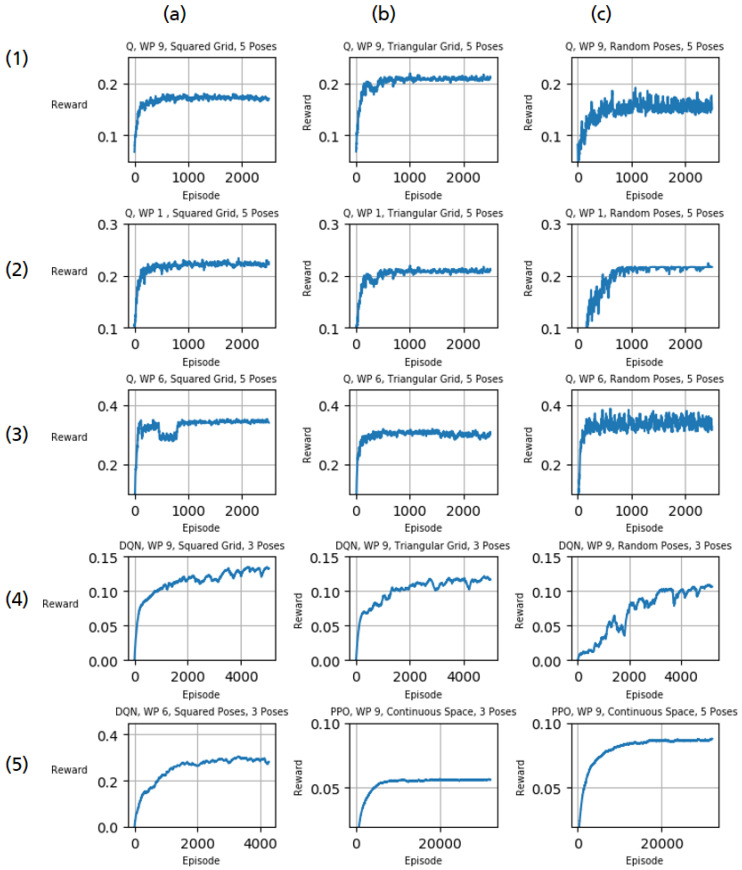
Training results of Q-learning, DQN and PPO using different action spaces (squared grid, triangular grid, random poses, or continuous) and trained on three different workpieces as denoted above each plot.

Figure 7 presents the learning process of our experiments with different parameters. The plot shows the reward (*y*-axis) for each episode (*x*-axis), which is equal to the percentage of covered surface area. For comparability, the inaccessible surface on the bottom side is subtracted. To provide a comprehensible picture, we smooth the reward per episode $R_{t}$ using an exponential moving average where the smoothing weight α∈[0,1).

(13) $$R_{t}=\left\{\begin{array}{cc} R_{t}, & \mathrm{if}\;t=0\\ \alpha R_{t-1}+\left(1-\alpha \right)\cdot R_{t}, & \mathrm{if}\;t>0 \end{array}\right.\;,$$

For workpiece 9, Q-learning achieved coverage of approximately 0.14 using a squared grid as action space and 5 view poses (Figure 7(1a)). In comparison, a triangular grid worked slightly better achieving coverage of about 0.165 (Figure 7(1b)). The training using randomly sampled view poses in the same workpiece converges more slowly and resulted in a slightly worse coverage of about 0.125 (Figure 7(1c)). Contrarily, a squared grid performed better than a triangular one for workpiece 1 and workpiece 6 achieving coverages of about 0.175 (Figure 7(2a)) and 0.26 (Figure 7(3a)) instead of 0.16 (Figure 7(2b)) and 0.24 (Figure 7(3b)), respectively. For workpiece 6, selecting random view poses lead to coverage of more than 0.28 (Figure 7(3c)). The same setup with workpiece 1 could not achieve this result exhibiting a coverage of about 0.17 (Figure 7(2c)).

In contrast to off-policy Q-learning, DQN requires more time for convergence, even though an episode is limited to 3 steps. On the other hand, the obtained results for workpiece 9 (Figure 7(4a–c)) indicate a better coverage ratio and are scalable. The result of DQN using a squared grid on workpiece 6 is shown in Figure 7(5a). Due to its sample-inefficient nature, PPO needs much more training samples. Even though the introduction of a continuous action space drastically increases the action space, PPO increased coverage to about 0.043 in case of three view poses per episode (Figure 7(5b)) and close to 0.07 using five view poses (Figure 7(5c)).

**Figure 8:**
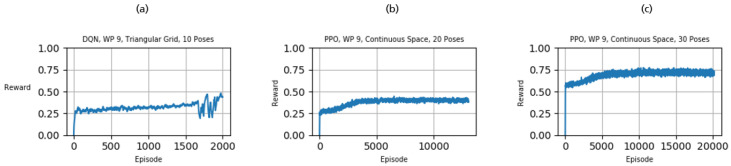
Training results of DQN and PPO aiming at a high coverage ratio.

Although these experiments prove the learning ability of the framework, they do not lead to complete coverage of the workpiece. Section 2.3.3 illustrated that Q-learning quickly becomes infeasible when increasing the number of possible actions. Therefore, we only considered DQN and PPO in the following experiments. Figure 8a shows the results for DQN learning to propose 10 view poses per episode. As indicated by the previous experiments, DQN can increase its performance accordingly and achieve coverage of approximately 0.5. Nevertheless, DQN suffers from limitations due to its discretized action space (see Section 2.3.3). Contrarily, PPO performs well when increasing the number of poses per episode up to 20 and 30 steps and reaches a coverage of more than 0.8 (Figure 8b,c)

The experiments are publicly available at https://github.com/christianlandgraf/rl_viewplanning (accessed on 12 March 2021) including training log, the view pose sequences, and trained models. Additionally, we provide a video illustrating the inspection setup including the robot kinematics and the 3D sensor as well as the accelerated training setup without robot kinematics. The video is available at https://www.youtube.com/watch?v=0mqPu0_qdD4 (accessed on 12 March 2021).

## 4. Discussion

Generally, our results demonstrate that the framework can increase the coverage of a specific number of view poses for all tested RL algorithms and workpieces. The performance of each introduced action space varies with the workpiece geometry. Further optimization and parameter tuning will improve the results in the future. The experiments provided in Section 3 serve as a proof of concept for the framework to plan automated inspection tasks in various settings.

Furthermore, the experiments do not guarantee the optimality of view poses, e.g., whether the algorithm is stuck in a local optimum. Instead, one might refer to the obtained results as *sub-optimal*. Nevertheless, we tuned the exploration factor and exploration discount to avoid local minima and solve the trade-off between exploration and exploitation. Although PPO performs slightly worse on a continuous action space than DQN and Q-learning on a discrete action space, it potentially outperforms these approaches in the future. For experiments shown in Figure 7(5b,c), the pitch angle remains fixed for simplicity. Therefore, point clouds are rendered from various poses, but with similar viewing directions. The shadowing of averted surfaces and sub-optimal view poses might cause the slightly worse performance of PPO.

An extended reward function may further increase learning performance towards optimal results. In our experiments, the reward solely depended on the scanned area concerning the workpiece’s total surface. Instead, one might aim at covering a certain degree of the total surface area instead of optimizing the reward regarding a fixed number of poses. Subsequently, an additional loss term might punish non-efficient poses.

Although the implemented surface area-based reward appears appropriate for finding view pose sets, it does not tackle the Traveling Salesman Problem (TSP) of the shortest trajectories between determined view poses. If the distance between view poses additionally alters the reward, the agent is theoretically capable of optimizing the order of view poses. However, combining VPP and TSP is likely to increase the required number of episodes significantly. Additionally, robot kinematics and collision avoidance need to be considered in the context of automated, robot-based inspection. Hence, alternative solutions to the TSP such as forwarding view poses obtained through the presented RL framework to independent solutions for robotic task sequencing problems might be more effective [32].

Besides the need for an improved reward function, we experienced another issue concerning the environment state space. To avoid approaching the same view pose multiple times per episode, the agent needs to get information about the episode’s previously scanned surface from the environment state. Unfortunately, the usage of raw point clouds as state representation is not applicable due to its size. At the moment, we bypassed this issue by avoiding the same pose to be executed twice per episode. Researchers of related fields realized the necessity for more compact and efficient representation of 3D point clouds early on [33]. Since lately, several deep learning techniques for obtaining meaningful binary descriptors for point clouds are available [34]. When adapted for the continuous RL state space, two point clouds rendered from adjacent poses are encoded to closely related, binary representations. All algorithms surveyed by [34] are capable of point cloud encoding. By extending the Adversarial Autoencoder (AAE) of [35] to accept 3D point clouds, the 3DAAE approach of [36] can reversely generate 3D shapes based on compact binary encodings.

Future work might include 3DAAE encodings of point clouds into state representation in the VPP framework to improve RL on continuous action/state spaces. Additionally, we intend to integrate more sophisticated action/state spaces and RL setups in general as well as other sensors, e.g., laser scanners.

## 5. Conclusions

The authors present a novel simulation framework for solving the view planning problem (VPP) for automated, robot-based inspection of workpieces. State-of-the-art Reinforcement Learning algorithms are deployed to determine suitable sensor view pose sets for a given CAD model. The framework allows the integration of any commonly used robot, sensor characteristics, and RL algorithm available in the OpenAI libraries. The experimental results for off-policy Q-learning, DQN, and PPO demonstrate the system’s ability to generate rational view poses for a given workpiece based on its position within the simulated robot cell. By considering robotic and sensor constraints, the approach significantly reduces required expert knowledge and manual programming for finding suitable view pose sets for inspection tasks. The framework builds on top of open-source libraries and is publicly available along with this article.

## Figures and Tables

**Table 1:** Overview of training settings. A detailed introduction of the training parameters is provided in Section 2.3.2. (**a**) Q-learning training parameters. (**b**) DQN training settings. (**c**) PPO training settings.

(a)	
Parameter	Value
Learning Rate (α)	0.1
Discount Factor (γ)	0.7
Initial Exploration Rate (ϵ)	0.9
Exploration Discount Factor	0.999
Number of Episodes	2500
(**b**)	
**Parameter**	**Value**
Policy	Multi-Layer Perceptron
	(2 layers with 64 neurons)
Learning Rate (α)	0.0001
Discount Factor (γ)	0.99
Initial Exploration Rate (ϵ)	0.9
Minimal Exploration Rate	0.05
Number of Episodes ($\epsilon_{\min}$)	20,000
Exploration Fraction of Training	0.2 (4000 episodes)
(**c**)	
**Parameter**	**Value**
Policy and Value Network	Multi-Layer Perceptron
	(2 layers with 64 neurons)
Learning Rate (α)	0.0001
Batch Size	4
Discount Factor (γ)	0.7
Clipping Range (ϵ)	0.2
Loss Entropy Coefficient ($c_{2}$)	0.1
Loss Value Function Coefficient ($c_{1}$)	0.5

## Data Availability

The data presented in this work are openly available together with the project code in https://github.com/christianlandgraf/rl_viewplanning (accessed on 12 March 2021). An example video is available at https://www.youtube.com/watch?v=0mqPu0_qdD4 (accessed on 12 March 2021).

## Abbreviations

The following abbreviations are used in this manuscript:

AAE	Adversarial Autoencoder
CAD	Computer-Aided Design
CD	Clearing Distance
CPP	Coverage Planning Problem
DQN	Deep Q-Networks
MDP	Markov Decision Process
MDPI	Multidisciplinary Digital Publishing Institute
OLP	Offline Programming
PCL	Point Cloud Library
POMDP	Partially Observable Markov Decision Process
PPO	Proximal Policy Optimization
RL	Reinforcement Learning
SARSA	State-Action Reward State-Action
TD	Temporal Difference
SCP	Set Cover Problem
TSP	Traveling Salesman Problem
VPP	View Planning Problem
WD	Working Distance
